# Three complete chloroplast genomes from two north American *Rhus* species and phylogenomics of Anacardiaceae

**DOI:** 10.1186/s12863-024-01200-6

**Published:** 2024-03-15

**Authors:** Lan Huang, Yujie Xu, Virginia Valcárcel, Sue Lutz, Jun Wen, Zhumei Ren

**Affiliations:** 1 School of Life Science, Shanxi University, 030006 Taiyuan, Shanxi China; 2 School of Geosciences, Qinghai Normal University, 810008 Xining, Qinghai China; 3 Departamento de Biología, Universidad Autónoma de Madrid, 28049 Madrid, Spain; 4 Centro de Investigación en Biodiversidady Cambio Global (CIBC-UAM) , Universidad Autónoma de Madrid, 28049 Madrid, Spain; 5 Department of Botany, National Museum of Natural History, Smithsonian Institution, 20013 Washington, DC USA

**Keywords:** Sumac, *Rhus*, Genome sequencing, Chloroplast phylogenomics

## Abstract

**Background:**

The suamc genus *Rhus* (sensu stricto) includes two subgenera, *Lobadium* (ca. 25 spp.) and *Rhus* (ca. 10 spp.). Their members, *R. glabra* and *R. typhina* (Rosanae: Sapindales: Anacardiaceae), are two economic important species. Chloroplast genome information is of great significance for the study of plant phylogeny and taxonomy.

**Results:**

The three complete chloroplast genomes from two *Rhus glabra* and one *R. typhina* accessions were obtained with a total of each about 159k bp in length including a large single-copy region (LSC, about 88k bp), a small single-copy regions (SSC, about 19k bp) and a pair of inverted repeats regions (IRa/IRb, about 26k bp), to form a canonical quadripartite structure. Each genome contained 88 protein-coding genes, 37 transfer RNA genes, eight ribosomal RNA genes and two pseudogenes. The overall GC content of the three genomes all were same (37.8%), and RSCU values showed that they all had the same codon prefers, i.e., to use codon ended with A/U (93%) except termination codon. Three variable hotspots, i.e., *ycf4*-*cemA*, *ndhF*-*rpl32*-*trnL* and *ccsA*-*ndhD*, and a total of 152–156 simple sequence repeats (SSR) were identified. The nonsynonymous (Ka)/synonymous (Ks) ratio was calculated, and *cemA* and *ycf2* genes are important indicators of gene evolution. The phylogenetic analyses of the family Anacardiaceae showed that the eight genera were grouped into three clusters, and supported the monophyly of the subfamilies and all the genera. The accessions of five *Rhus* species formed four clusters, while, one individual of *R. typhina* grouped with the *R. glabra* accessions instead of clustering into the two other individuals of *R. typhina* in the subgenus *Rhus*, which showed a paraphyletic relationship.

**Conclusions:**

Comparing the complete chloroplast genomes of the *Rhus* species, it was found that most SSRs were A/T rich and located in the intergenic spacer, and the nucleotide divergence exhibited higher levels in the non-coding region than in the coding region. The Ka/Ks ratio of *cemA* gene was > 1 for species collected in America, while it was < 1 for other species in China, which dedicated that the *Rhus* species from North America and East Asia have different evolutionary pressure. The phylogenetic analysis of the complete chloroplast genome clarified the *Rhus* placement and relationship. The results obtained in this study are expected to provide valuable genetic resources to perform species identification, molecular breeding, and intraspecific diversity of the *Rhus* species.

**Supplementary Information:**

The online version contains supplementary material available at 10.1186/s12863-024-01200-6.

## Background

The sumac genus *Rhus* L. is the largest and most widespread genus in the family Anacardiaceae, within which two subfamilies Anacardioideae and Spondioideae were recognized, with a disjunct distribution that spans over temperate and subtropical latitudes in the Northern Hemisphere [1–5]. In general, sumac can grow in nonagriculturally viable regions, and various species have been used by indigenous cultures for medicinal and other purposes, suggesting potential for commercializing the bioactivity of these plants [6]. Different parts of *Rhus* plants can be used in medicine and food, and species of the genus have also been utilized in soil conservation and environmental restoration [7–10].

The representative species, including *R. coriaria* (tanner’s sumac), *R. copallina* (winged or shining sumac), *R. glabra* (smooth sumac), *R. undulate* (Kuni bush), and *R. verniciflua* (Japanese sumac), are native to Mediterranean Basin, Eastern North America, Western North America, South Africa and Asia, respectively [11]. Specially, only the two species *R. glabra* and *R. typhina* are the host plant species of the *Rhus*-gall aphids *Melaphis* [12]. *R. typhina* were introduced to China in 1959 [13], that is considered potentially invasive in its non-native habitats [14]. The branches of *R. glabra* have antimicrobial activity, which is often used in folk medicine by North American native people [7]. As a summary, *R. glabra* and *R. typhina* are not only an important species for the landscape of certain open habitats but also ecologically essential as one of the two hosts of the monotypic *Rhus*-gall aphids *Melaphis* as well as a medicinal plant.

The genus *Rhus* (sensu stricto) is classified into two subgenera: subgenus *Rhus* and subgenus *Lobadium* [15]. *Rhus* subgenus includes 10 species and is characterized by the deciduous and imparipinnately compound leaves, the flowers appearing after the leaves, and inflorescences as terminal thyrses, which are subtended by deciduous bracts [15, 16]. The phylogenetic relationship of the genus *Rhus* was analyzed using a few number of nuclear and chloroplast regions with a limit sampling, and the results based on ITS sequence indicated that the subgenus *Rhus* was nested into *Lobadium* to form a paraphyletic group, but *R. glabra* is not included in this study [4]. The analysis of combined data sets, i.e., *ITS*, *trnL*-*trnF*, *ndhF*; *Nia*-*i3*, *trnC*-*trnD*, indicated that subgenus *Rhus* is monophyletic and *R. glabra* is sister with *R*. *typhina* [17–19], and the *trnC*-*trnD* region provided slightly more parsimony-informative characters than the *ndhF* gene and *trnL*-*trnF* region [18].

The chloroplast genome is a valuable resource in molecular phylogenetic analysis [20, 21], and contains a pair of inverted repeat (IR) regions separated by a large single copy (LSC) and a small single copy (SSC) region [22, 23]. This quadripartite structure is highly conserved in gene content and genome organization relative to the plant nuclear and mitochondrial genomes [23]. Comparative analyses between chloroplast genomes of plant species revealed structural variations, such as IR or gene loss that are considered as a result of environmental adaptation [23]. Despite the plastid genome generally has a slower evolutionary rate than the nuclear genome, it is frequently used in phylogenetic studies of plants [24, 25]. This is mostly due to the fact of its uniparental inheritance, which provides unique information about the evolutionary history of the group under study [26]. More recently, it is considered that the chloroplast genome data could authenticate evolutionary relationships and confirm phylogenetic classifications for plants at the family and genus level [27]. The chloroplast genome hence has been widely utilized as a good marker for the phylogenetic reconstructions of plants at species, genus and family levels.

In this study, we sequenced three complete chloroplast genomes of *Rhus glabra* and *R. typhina* using the high throughout sequencing method on an Illumina HiSeq 4000 platform. We aimed to characterize the structure and organization of the *R. glabra* and *R. typhina* chloroplast genomes and the nucleotide divergence in genus *Rhus*, and conduct an initial chloroplast phylogenomic analysis in the family Anacardiceae, with an emphasis of *Rhus* subgenus *Rhus*.

## Results

### General characteristics of three chloroplast genome of ***Rhus*** species

The two complete chloroplast genomes of *Rhus glabra* were assembled as a total of 159,984 bp and 159,944 bp, and *R. typhina* is 159,940 bp in length (Table 1). The nucleotide composition of the genome were calculated to be 31.40% T, 30.80% A, 19.20% C, and 18.60% G, respectively. By BLAST and Finding Repeat Region in Geneious software, the complete chloroplast genomes were divided into a canonical quadripartite structure with a large single-copy region (LSC, 87,904 − 88,002 bp), a small single-copy region (SSC, 18,862 − 18,891 bp) and a pair of inverted repeats regions (IRa/IRb, 26,559 − 26,560 bp). The GC content of the LSC, SSC and IR regions were 35.8%, 32.4% and 42.9%, respectively. The complete chloroplast genomes of two *R. glabra* individuals (Voucher nos. Ren_P3002 and Ren_P3051) and one *R. typhina* (Voucher no. Ren_P3053) with gene annotations were submitted to GenBank under the accession numbers OR800752, OR800753 and OR773067, respectively.

**Table 1:** Features of *Rhus* chloroplast genomes used in this study

Species	*R. glabra*		*R. typhina*			*R. chinensis*	*R*.* potaninii*		*R*. *punjabensis*
Accession No.	OR800752	OR800753	OR773067	MN866894	MT083895	OP326720	MT230556	MN866893	MT230555
Location	Ohio, US	Georgia, US	New York, US	Unknown	Shandong, China	Hubei, China	Shaanxi, China	Beijing, China	Hubei, China
Total Length (bp)	159,984	159,944	159,940	160,254	160,204	159,187	159,620	159,616	159,617
LSC (bp)	88,002	87,935	87,955	87,789	87,789	87,653	87,722	87,710	87,694
SSC (bp)	18,862	18,891	18,865	19,453	19,319	18,522	18,948	18,956	18,971
IR (bp)	26,560	26,559	26,560	26,506	26,548	26,506	26,475	26,475	26,476
Total Genes	135		135			133	133		133
CDS	88		88			86	86		86
tRNA	37		37			37	37		37
rRNA	8		8			8	8		8
Pseudogene	*infA*, *ycf1*		*infA, ycf1*			*infA, ycf1*	*infA*, *ycf1*		*infA*, *ycf1*
Total GC%	37.8		37.8			37.8	37.9		37.9
LSC (GC%)	35.8		35.8	35.8	35.8	35.9	36.0		36.0
SSC (GC%)	32.4		32.4	32.6	32.5	32.6	32.6		32.6
IR (GC%)	42.9		42.9	42.9	42.9	42.6	43.0		43.0
A%	30.80		30.80	30.85	30.80	30.80	30.72	30.72	30.72
C%	19.20		19.20	19.21	19.20	19.30	19.29	19.30	19.29
G%	18.60		18.60	18.57	18.60	18.60	18.61	18.60	18.61
T%	31.40		31.40	31.37	31.40	31.40	31.38	31.38	31.38

**Fig. 1:**
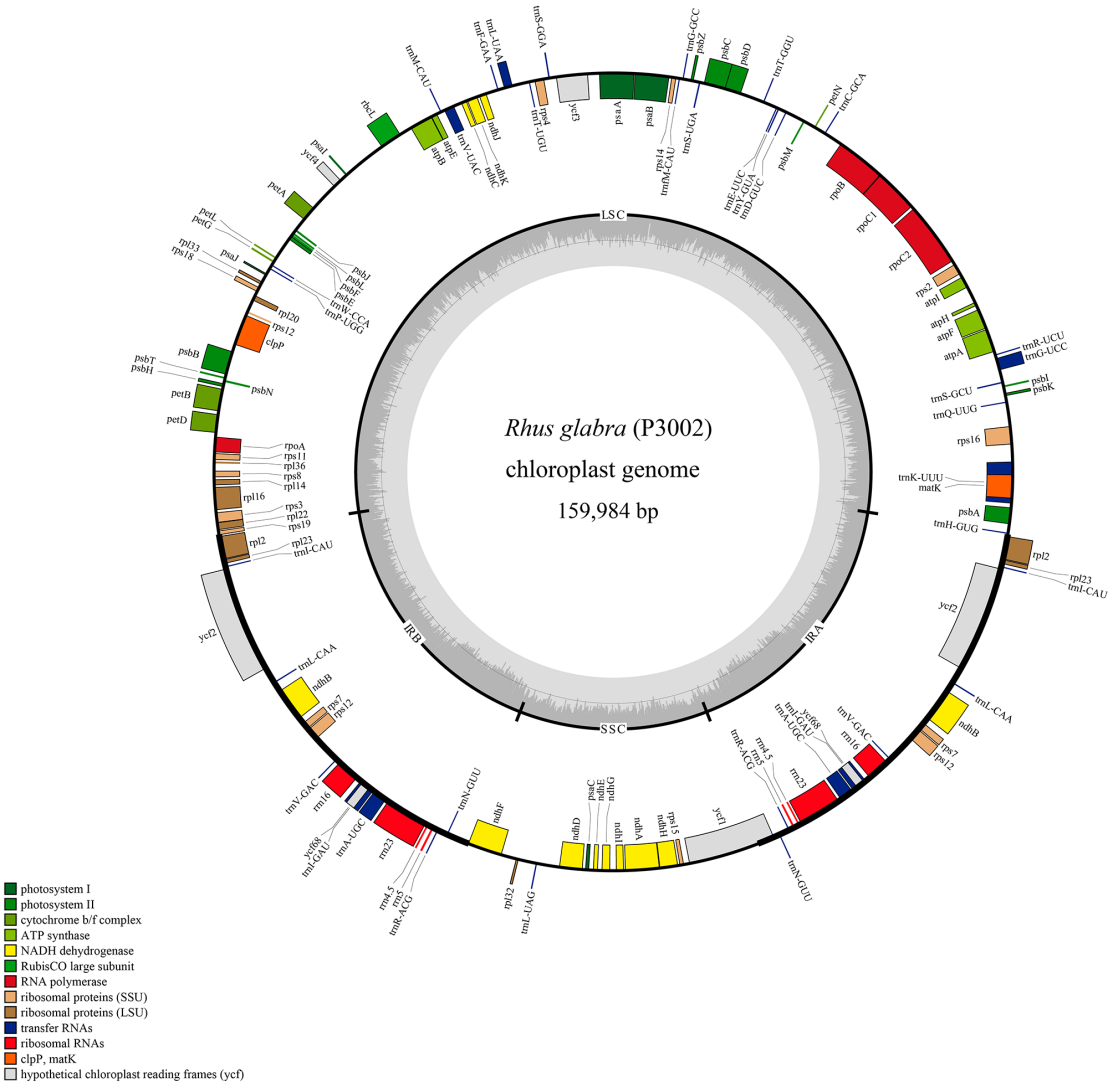
Chloroplast genome map of *Rhus glabra* (Accession No. OR800752). Genes encoded in the forward direction are located on the outside of the ring, while those encoded in the reverse direction on the inside of the ring. The gray circles inside represent the GC content

A total of 135 genes in the three chloroplast genomes were both annotated, including 88 protein-coding genes, eight ribosomal RNA genes, 37 transfer RNA genes and two pseudogenes (Table 1). The classifications of the 135 genes in the *Rhus glabra* complete chloroplast genome were showed in Table 2, which were classified into five categories. Based on the analysis on the protein-coding genes, we found that 88 protein-coding genes were dispersedly located in quadripartite structure, i.e., 60 protein-coding genes in LSC region, 12 in SSC region and eight in IR region (Fig. 1, Fig. S1 and Fig. S2). Four rRNAs, i.e., *rrn16*, *rrn23*, *rrn4.5* and *rrn5*, were only located in a pair of inverted repeats regions, and appeared symmetrical distribution. In the 37 tRNA genes, *trnL-UAG* was only one which was harbored in SSC, while 14 tRNA genes appeared symmetrically in IRs and the remains in LSC.

**Table 2:** Gene classification in the chloroplast genome of *Rhus* genus

Category of genes (No.)	Group (No.)	Name of gene (No. of duplicated gene)
Self replication (74)	rRNA (8)	*rrn4.5*(2),*rrn5*(2),*rrn16*(2),*rrn23*(2)
	tRNA (37)	*trnH-GUG*,*trnK-UUU*,*trnQ-UUG*,*trnS-GCU*,*trnG-UCC*,*trnR-UCU*,*trnC-GCA*,*trnD-GUC*,*trnY-GUA,trnE-UUC*,*trnT-GGU*,*trnS-UGA*,*trnG-UCC*,*trnfM-CAU*,*trnS-GGA*,*trnT-UGU*,*trnL-UAA*,*trnF-GAA*,*trnV-UAG*,*trnM-CAU*,*trnW-CCA*,*trnP-UGG*,*trnI-CAU*(2),*trnL-CAA*(2),*trnV-GAC*(2),*trnI-GAU*(2),*trnA-UGC*(2),*trnR-ACG*(2),*trnN-GUU*(2),*trnL-UAG*
	Small subunit of ribosome (14)	rps16*,*rps2*,*rps14*,*rps4*,*rps18*,*rps12*(2)*,*rps11*,*rps*8,*rps*3,*rps19*,*rps7*(2),*rps15*
	Large subunit of ribosome (11)	*rpl33*,*rpl20*,*rpl36*,*rpl14*,rpl16*,*rpl22*,*rpl2*(2)*,*rpl23*(2),*rpl32*
	DNA dependent RNA polymerase (4)	*rpoC2*,rpoC1*,*rpoB*,*rpoA*
Genes for photosynthesis (46)	Subunits of photosystem I (5)	*psaB*,*psaA*,*psaI*,*psaJ*,*psaC*
	Subunits of photosystem II (15)	*psbA*,*psbK*,*psbI*,*psbM*,*psbD*,*psbC*,*pzbZ*,*psbJ*,*psbL*,*psbF*,*psbB*,*psbT*,*psbN*,*psbH*,*psbE*
	Subunits of cytochrome b/f complex (6)	petD*,petB*,*petG*,*petL*,*petA*,*petN*
	Subunits of ATP synthase (6)	*atpA*,atpF*,*atpH*,*atpI*,*atpE*,*atpB*
	Subunits of NADH synthase (12)	*ndhJ*,*ndhK*,*ndhC*,*ndhB*(2)*,*ndhF*,*ndhD*,*ndhE*,*ndhG*,*ndhI*,ndhA*,*ndhH*
	Subunits of rubisco (1)	*rbcL*
	Subunits P (1)	clpP**
Other genes (5)	Mature (1)	*matK*
	Envelop membrance protein (1)	*cemA*
	Subunit of acetyl-CoA carboxylase (1)	*accD*
	C-type cytochrome synthesis (1)	*ccsA*
	Translational initiation factor (1)	*infA*
Genes of unknown function (10)	Conserved open reading frames (10)	*ycf1*(2),*ycf2*(2),ycf3**,*ycf4*,*ycf15*(2),ycf68(2)

Notes: * represent gene with one intron and ** represent gene with two introns

In case of intron-containing genes, there were 17 intron-containing genes including one gene (*ndhA*) in the SSC, 12 genes in the LSC and four genes (*rpl2*, *ndhB*, *trnI-GAU*, *trnA-UGC*) duplicated in the IR. The two genes *ycf3* and *clpP* were located in the LSC with possessing two introns (Table 2 and Table S1). The gene *trnL*-*UAA* possessed the shortest intron with 469 bp in length, wihle the longest intron was founded in *trnK*-*UUU* with 2598 bp in length, in which the the protein-coding gene *matK* was inserted, which is the general characteristics in plants [28]. The gene *rps12* was trans-spliced with the duplicated 3′ end in the IRs and the 5′ end located in the LSC region, as previously reported in other plants [29].

### Codon usage of protein-coding genes

The values of RSCU of 88 protein-coding genes of two *Rhus glabra* chloroplast genomes (accession Nos.OR800752 and OR800753) and three *R. typhina* accessions (accession Nos. MN866894, MT083895 and OR773067) from GenBank were displayed in Table S2. All the samples had the same condon bias for each amino acid. The protein-coding gene totally contained 26,700 − 26,807 codons (including 88 terminal codons) in different individuals, among which Leu was the most aboudant amino acid, with about 2816 (10.51%) − 2824 (10.53%), while Cys was the least, with 313 (1.18%) − 316 (1.18%), respectively. In all the termination codons, UAA is the most frequently used amino acid. The RSCU values of 30 codons were all > 1 except termination codon, in which codon ended with A/U was 93% and codon ended with C/G was 7%, indicating that these codons tended to end in A/U. The codon usage is extremely conserved in the these species due to as species belong to the genus level conservation, which was consistent with the previous reports on the many land plants’ chloroplast genomes [30, 31].

### SSR analysis

**Fig. 2:**
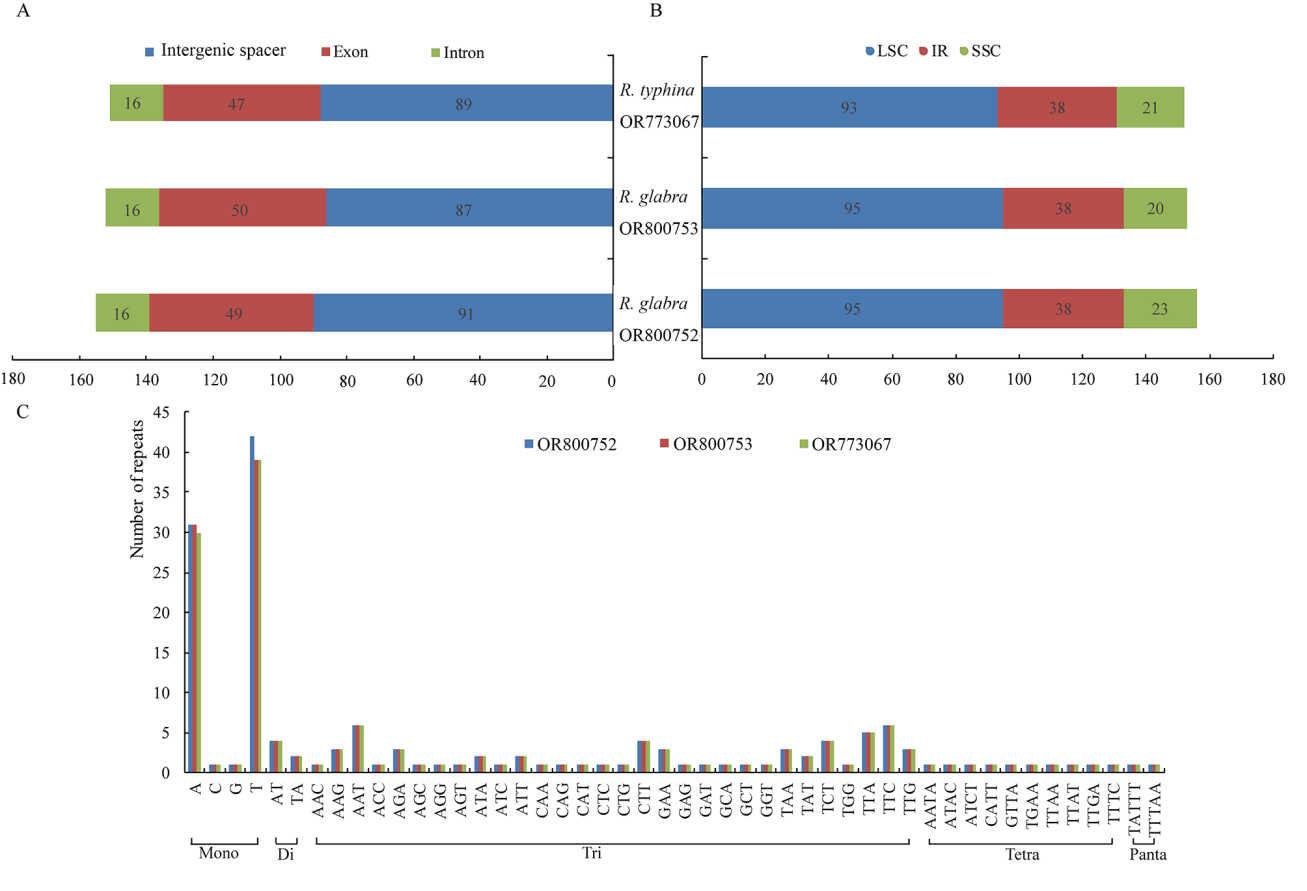
The type and distribution of SSRs in the three *Rhus* chloroplast genomes. **A**: Proportion of SSR distribution in the intergenic spacer, exon and intron; **B**: Frequency of SSR occurrence in the LSC, SSC, and IR region; **C**: SSR repeat types and numbers

SSR loci were detected in three chloroplast genomes, and a total of 152, 153 and 156 SSRs were in one *R. typhina* individual (accession No. OR773067) and two *R. glabra* accessions (accession Nos. OR800752 and OR800753), respectively. The SSR distribution in different gene regions and genomic quadripartite structures were shown in Fig. 2. The three samples have the same amounts of repeat types (six dinucleotides, 63 trinucleotides, 10 tetranucleotides, and two pentanucleotides) except mononucleotide type containing 71, 72 and 75, separately (Fig. 2C). Regard to each individuals, majority of SSRs focused on mononucleotide (47 − 48%) and trinucleotide (40 − 41%) in Fig. 2C. Nearly all mononucleotide SSRs were composed of A/T (97%), and trinucleotide repaets of AAT and TTC were the second most common SSRs in these three chloroplast genomes (Fig. 2C).

### Contraction and expansion of IR region

**Fig. 3:**
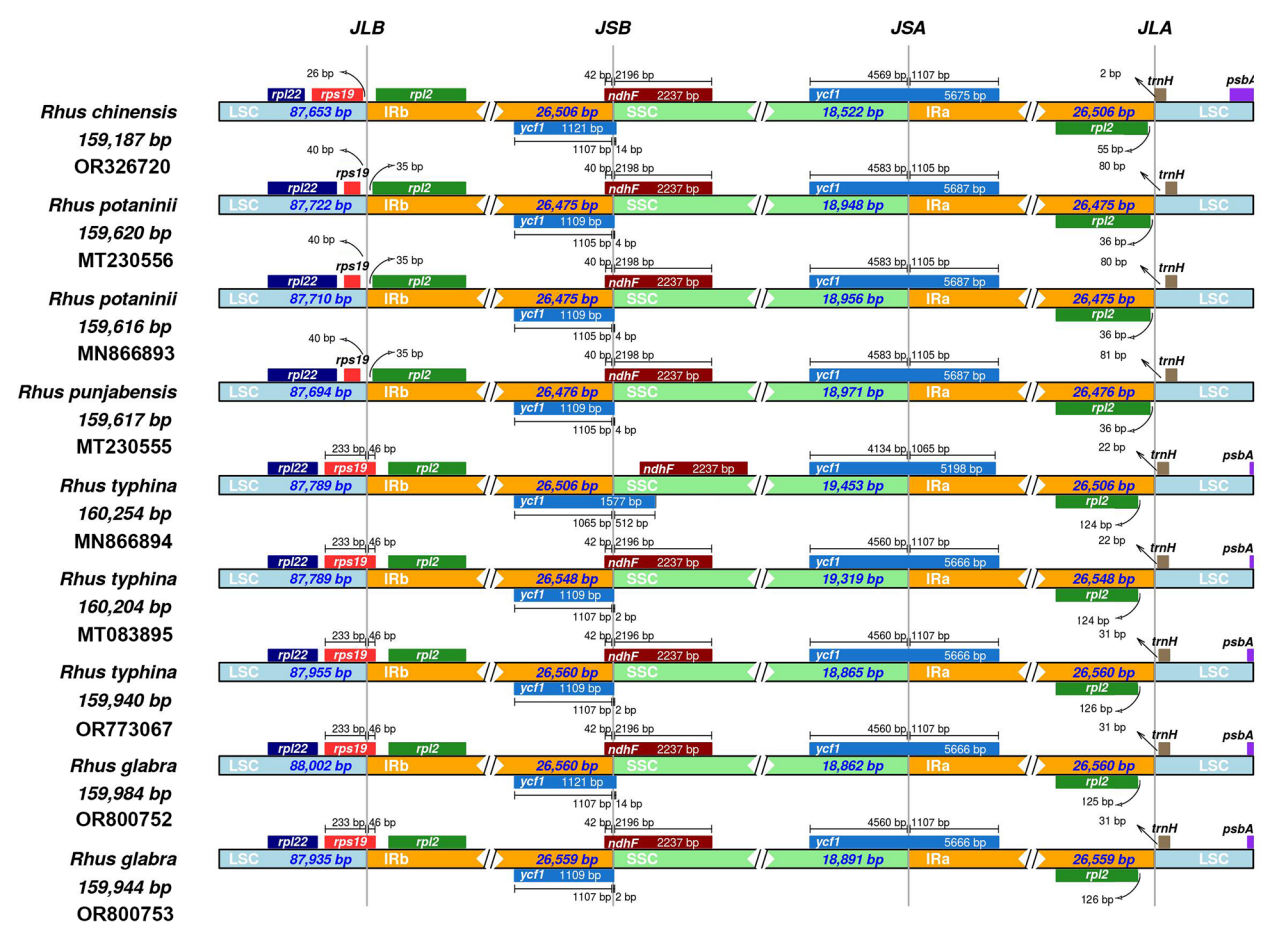
Comparison of the borders of the LSC, SSC and IR regions in chloroplast genomes of *Rhus.* The junction of these parts, i.e., JLB (junction of LSC and IRb), JSB (junction of SSC and IRb), JSA (junction of SSC and IRa) and JLA (junction of LSC and IRa)

Six chloroplast genome sequences of *Rhus* species in GenBank (Accession Nos. OP326720, MT230556, MN866893, MT230555, MN866894 and MT083895) were compared with three chloroplast genomes present in this study. To analyze the boundary and detect gene contraction and expansion of chloroplast sequences, IRscope software was used to visualize the junction of single copy and the inverted repeat regions, shown in Fig. 3. The length of IR regions ranged from 26,475 to 26,560 bp, and the region of IR/LSC and IR/SSC junction in these *Rhus* species showed the same boundary genes, i.e., *rps19*, *rpl2*, *ycf1*, *ndhF*, *ycf1*, *trnN*, *rpl2* and *trnH*. The two protein-coding genes *ndhF* and *ycf1* crossed the IRb/SSC and SSC/IRa junction in all the genomes, respectively. Furthermore, *ycf1* gene was partially duplicated at the IRb/SSC boundary, resulting in a pseudogene, which can be obversied in nine *Rhus* genomes. In four accessions (Accession Nos. OP326720, MN866893, MT230556 and MT230555), the JLB boundary was 26 and 40 bp away from *rps19*, while in the other five accessions species the *rps19* gene crossed the JLB boundary. In addition, the pseudogene *ycf1* usually located at the end of IRb region crossing SSC with less than 14 bp, while it is very special, that is, in *R*. *typhina* (MN866894), *ycf1* pseudogene crossed JSB with 512 bp into SSC region, and overlapped with the *ndhF* gene in SSC region with a stretch of 45 bp. The *Rhus* chloroplast genomes are generally conserved, but slightly with a variaton of either expansion or contraction of the single copy and IR boundary regions.

### Sequence divergence and hotspots

**Fig. 4:**
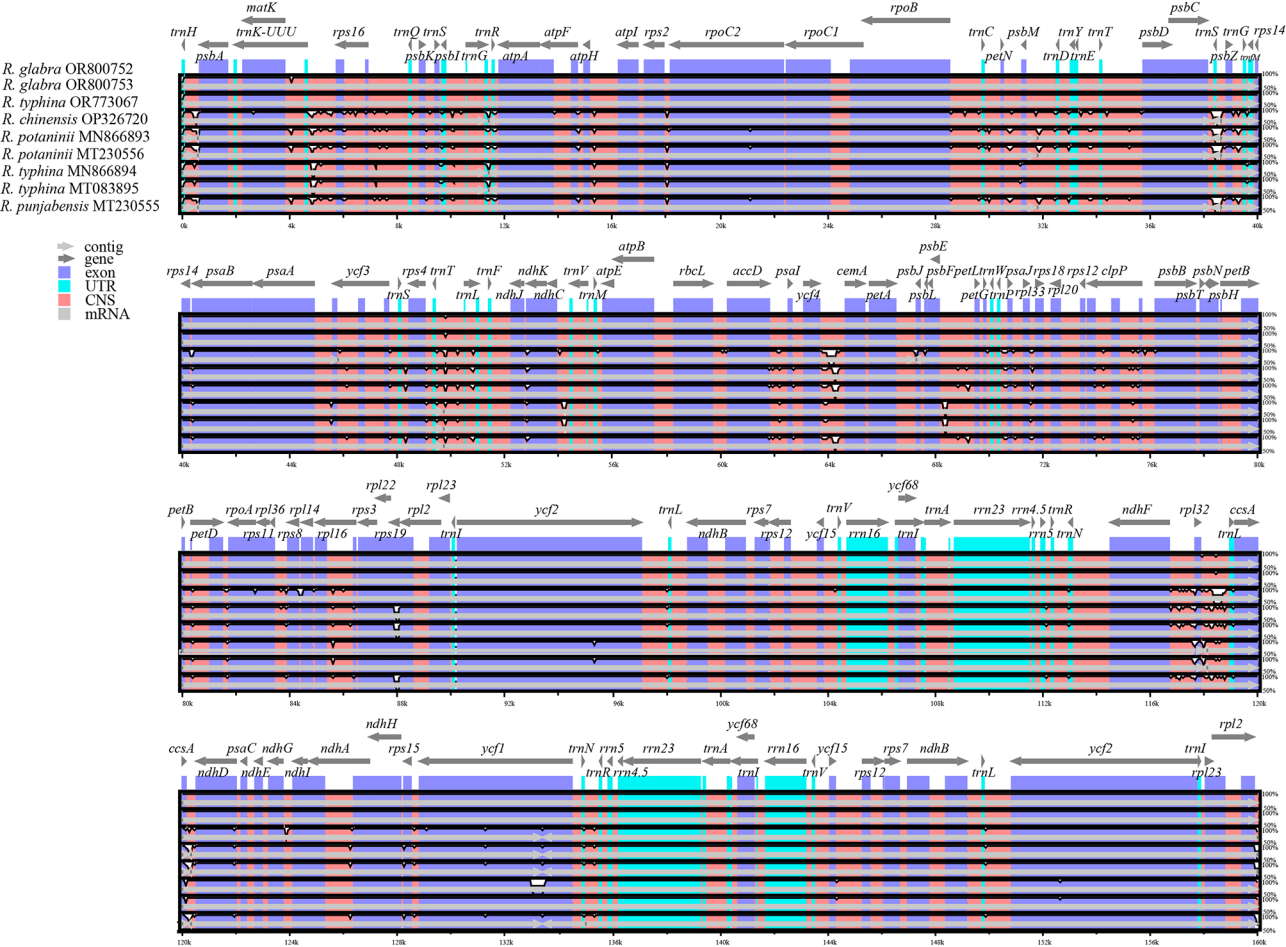
Visualization map of genome alignment of the chloroplast genomes using *Rhus glabra* (OR800752) as a reference by mVISTA. X-axis: the coordinates in chloroplast genomes; Y-axis: the average percent identity (50–100%)

We determined the genome divergence of *Rhus* genus by genome alignment using the program mVISTA, and the visualization map was shown in Fig. 4. It was showed that the intraspecific variation of each species was low, and the exons were extremely conservative, while the interspecific variation was high, and the divergence regions were mainly from these ones, i.e., *trnH*-*psbA*, *trnK*-*rps16*, *ycf4*-*cemA*, *rps19*, *ndhF*-*rpl32*-*trnL*, *trnS*-*psbZ* and *ccsA*-*ndhD* regions, majority of which happened in introns and intergenic spacer.

**Fig. 5:**
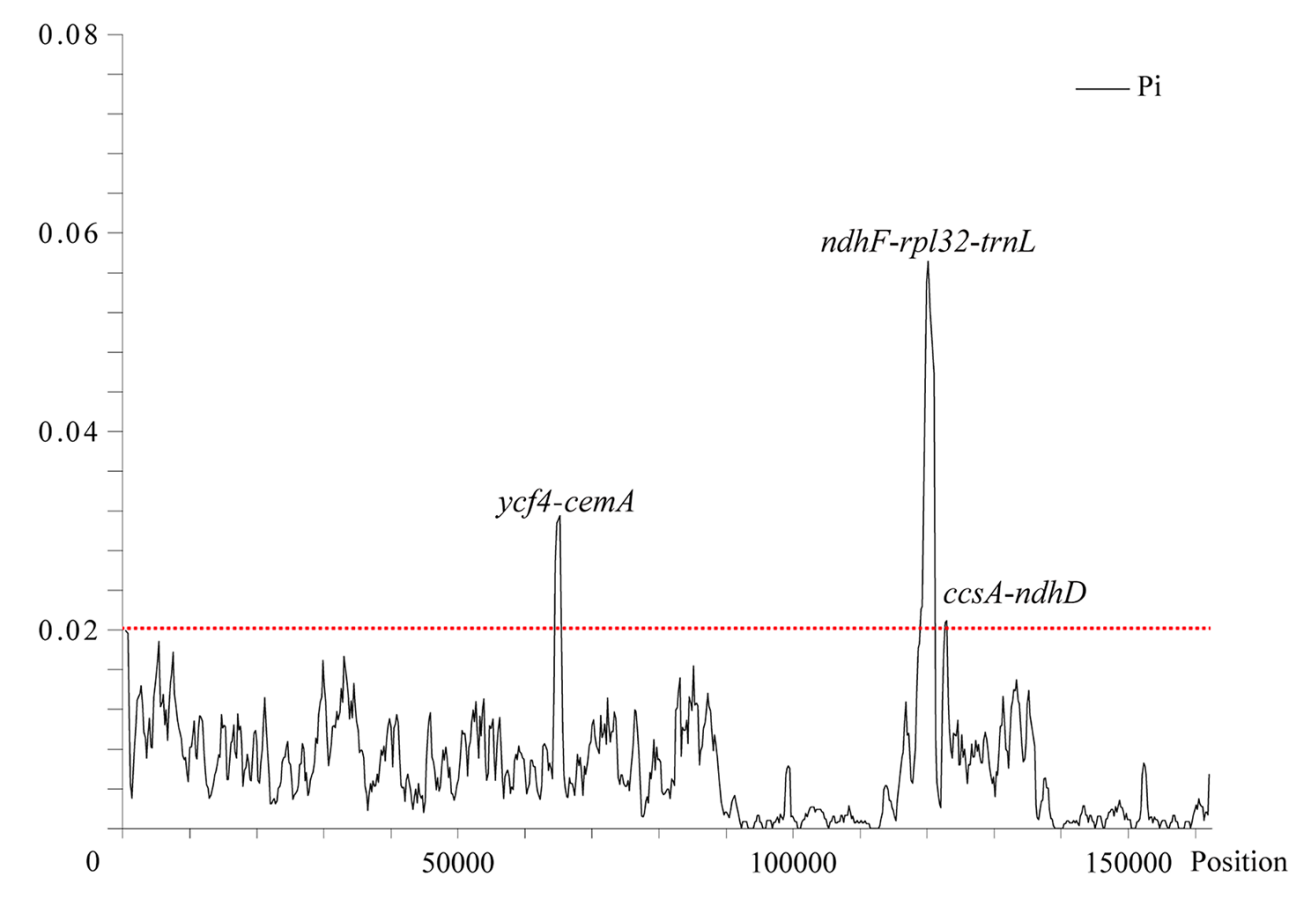
Sliding-window analysis of the chloroplast genomes in *Rhus* genus. Window length: 800 bp; step size: 200 bp; X-axis: position of the midpoint of a window; Y-axis: nucleotide diversity of each window

We also identified the mutational hotspots of the chloroplast genomes of the *Rhus* species in Fig. 5. There were three positions (*ycf4*-*cemA*, *ndhF*-*rpl32*-*trnL* and *ccsA*-*ndhD*) in the nine *Rhus* species exhibited high nucleotide diversity (Pi values > 0.02) tested by the DNaSP software, among which the average Pi value of the *ccsA*-*ndhD* region was the highest (Pi values = 0.03819) in Table 3.

**Table 3:** Variation of complete chloroplast genomes, potential molecular markers and universal barcodes

Regions	Length	Variable sites		Parsimony information sites		Nucleotide diversity
		numbers	%	numbers	%	
Two *R. glabra* genomes	160,022	23	0.0144	0	0	0.00014
Three *R. typhina* genomes	161,410	430	0.2664	0	0	0.00181
*trnH*-psbA*	613	43	7.0147	16	2.6101	0.03109
*trnK*-rps16*	1177	36	3.0586	29	2.4639	0.01789
*ycf4*-cemA**	982	66	6.7210	29	2.9532	0.03528
*ndhF*-*rpl32*-trnL**	2849	153	5.3703	127	4.4577	0.03399
*ccsA*-ndhD**	419	29	6.9212	23	5.4893	0.03819
rps19*	322	1	0.3106	0	0	0.00181
trnS-psbZ*	375	4	1.0667	4	1.0667	0.00573
trnC-D*	2772	65	2.3449	51	1.8398	0.01126
trnL-F*	370	13	3.5135	9	2.4324	0.01547

Notes: *represent universal barcodes and **represent potential molecular markers

Additionally, we calculated the nucleotide diversity between two *R. glabra* and three *R. typhina* chloroplast genomes (Table 3). *R. glabra* and *R. typhina* chloroplast genomes were aligned with a matrix of 160,022 bp and 161,410 bp, respectively. Twenty three and 430 variable sites were examined, respectively, and intraspecific difference of *R. typhina* is located on *rpl32* gene (Fig. S3). The pair variable sites between the *R. glabra* and *R. typhina* individuals indicated that the variation (about 0.27%) of the *R. typhina* individual (accession No. OR773067) and the other two *R. typhina* accessions (Nos. MN866894 and MT083895) was much higher than that (about 0.01%) with the two *R. glabra* accessions (Nos. OR800752, OR800753).

### Synonymous (Ks) and nonsynonymous (Ka) substitution rate analyses

**Fig. 6:**
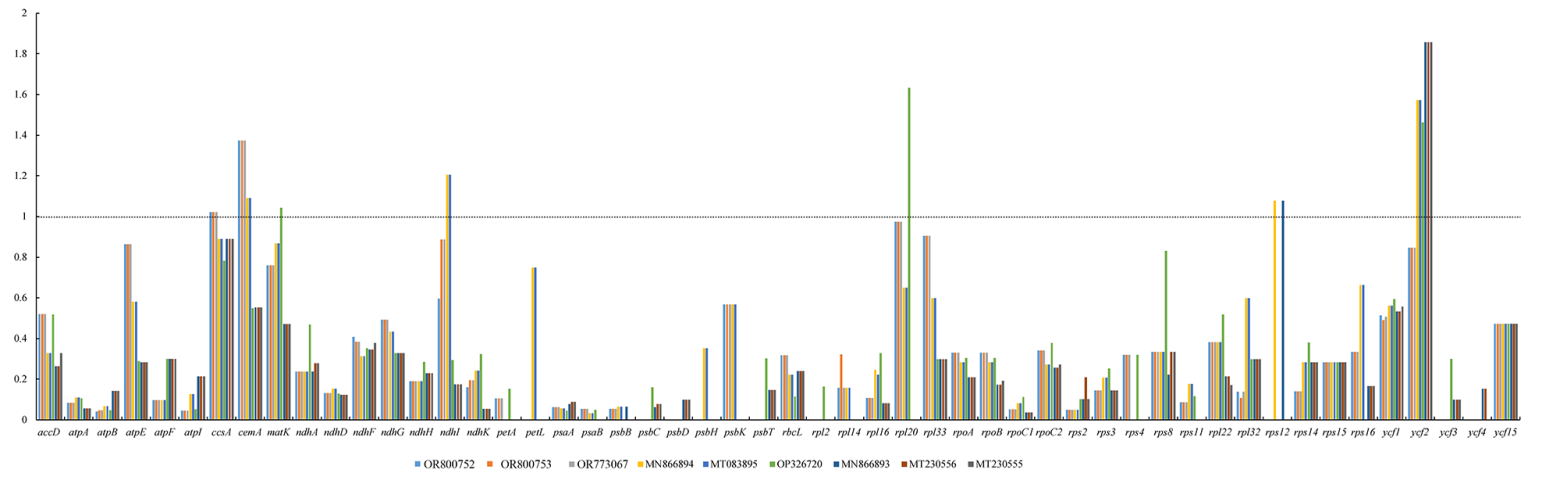
The Ka/Ks ratios of 78 protein-coding genes in the *Rhus* genus taking *Pistacia chinensis* (MK738124) as a reference

We annotated 88 protein-coding genes in our samples of *R. typhina* and *R. glabra*, while, there are 86 genes annotated in the *Rhus* accessions from GenBank. So we used the common 86 protein-coding genes in *Rhus* genus, and meanwhile excluded the eight repeat genes in the IR region, that is, pairwise comparisons of 78 commom protein-coding genes in all *Rhus* species were finally employed to calculate Ka and Ks substitution rates as compared with *Pistacia chinensis* (MK738124). The genes with Ka/Ks values > 0 were shown in Fig. 6, indicating nucleotide substututions of different protein-coding genes are different. Among the genes with Ka/Ks ratio > 1, *cemA* gene for American species (our samples, accession Nos. OR800752, OR800753 and OR773067) and *R. typhina* (accession Nos. MN866894 and MT083895) were undergoing positive selection pressure, while Ka/Ks ratio of *cemA* gene < 1 for other species, which were undergoing purifying selection. Moreovere, the *ycf2* gene was different from the above genes with Ka/Ks ratio < 1 in American species, while > 1 in other accessions. The genes with the ratio of Ka/Ks = 0 or NA (Ks = 0) didn’t be displayed in Fig. 6, including 27 protein-coding genes, i.e., *atpH*, *clpP*, *ndhB*, *ndhC*, *ndhE*, *petB*, *petD*, *petG*, *petN*, *psbN*, *psaC*, *psaJ*, *psaI*, *psbA*, *psbE*, *psbF*, *psbI*, *psbJ*, *psbL*, *psbM*, *psbN*, *psbZ*, *rpl36*, *rps7*, *rpl23*, *rps18, rps19*, which dedicated that these genes were the most conserved genes and nucleotide substututions were slow.

### Phylogenetic analysis

**Fig. 7:**
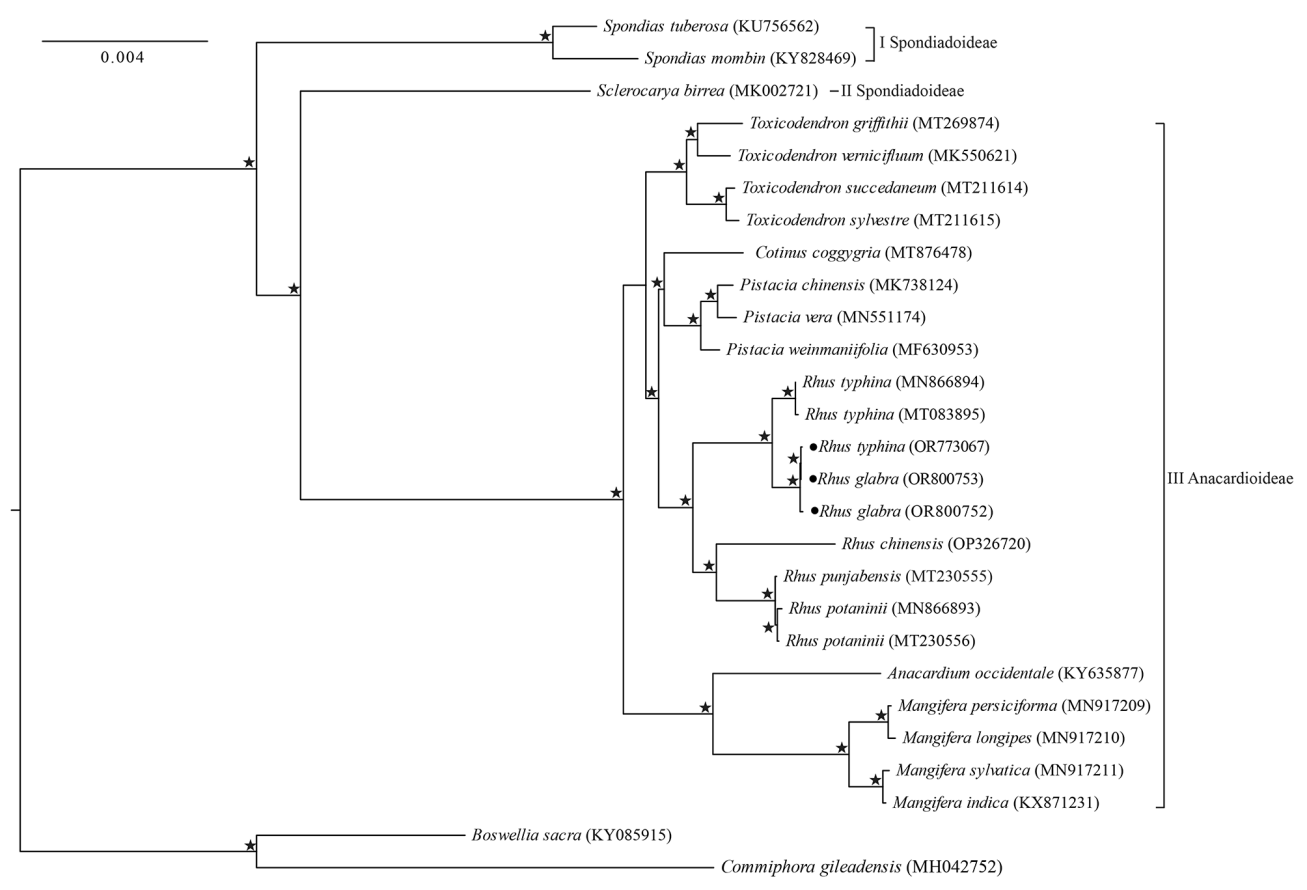
Maximum likelihood (ML) tree constructed by protein-coding genes of the Anarcadiceae chloroplast genomes. Stars represent nodes with 100% bootstrap values. Dots represent three *Rhus* individuals in this study

The phylogenetic tree of the 25 Anacardiaceae species supported the monophyly of each genus, and provided high support for the phylogenetic relationships among genera (Fig. 7). Three well-supported clades were identified: (I) *Spondias* of Spondiadoideae, (II) *Sclerocarya* of Spondiadoideae, and the remaining six genera (III) of Anacardioideae. The genus *Spondias* was sister to the clade of the remaining genera with 100% support. Within subfamily Anacardioideae, *Anacardium* and *Mangifera* consitituted a clade; *Rhus* is sister to the *Cotinus* + *Pistacia* clade; and *Toxicodendron* is then sister to the clade of (*Rhus*, (*Cotinus*, *Pistacia*)). In *Rhus* genus, *R. potaninii* was sister to *R. punjabensis*, then grouped with *R. chinensis*; the different individuals from the sister species *R. glabra* and *R. typhina* formed two groups with paraphyletic relationship: one in two *R. typhina* individuals (accession Nos. MN866894 and MT083895) from China, and the other one is two *R. glabra* individuals (accession Nos. OR800752 and OR800753) and one *R. typhina* individual (accession No. OR773067) from US.

## Discussion

Overall, the *Rhus* chloroplast genomes were relatively conserved, including the genomes structures, nucleotide composition, gene orders, G + C contents, and codon usages, which are both a canonical quadripartite structure: a large single-copy region (LSC, about 88k bp), a small single-copy regions (SSC, about 19k bp) and a pair of inverted repeats regions (IRa/IRb, about 26k bp). Each genome contained 88 protein-coding genes, 37 transfer RNAs genes, eight ribosomal RNA genes and two pseudogenes. The total GC contents (37.8%) of three *Rhus* individuals were almost the same as those of other Anacardiaceae species, e.g., *R. potaninii* (37.9%) [2], *R. chinensis* (37.8%) [28], *R. punjabensis* (37.9%) and *R. typhina* (37.8%). The GC content of IR region in all *Rhus* genus is the highest, possibly due to the presence of four extremely conserved rRNA genes in IR region, which had high GC contents.

The codon preference in different species is different and has racial specificity [32, 33]. The relative synonymous codon usage (RSCU) is one of the commonly used parameters to measure codon usage bias [33]. The RSCU values of 30 codons were all > 1 except termination codon in two *R. glabra* and three *R. typhina* individuals, in which codon ended with A/U was 93% and is similar to the plants of the same genus [30] and other plants, such as *Cinnamomum camphora* [34], *Wurfbainia villosa* [35] and *Aconitum hemsleyanum* [36]. Those were consistent with the analysis that dicotyledons’ codon tend to end in A/T [37].

It was reported that simple sequence repeats (SSRs), or microsatellites, are repeat sequences of typically 1–6 bp that are distributed throughout the genome [34, 36]. Compared with other neutral DNA regions, SSRs usually have a higher mutation rate due to slipped DNA strands and have been used for the study of population genetics, evolutionary and ecological studies [38, 39]. We looked for SSRs of 10 bp or longer, as these have been suggested to be prone to slipped strand mispairing and believed to be the main mutational mechanism for SSR polymorphisms [39]. In *Rhus* chloroplast genomes, five types were detected and majority of SSRs focused on mononucleotide and trinucleotide, which were consistent with the previous observation that the SSRs of chloroplast genomes are dominated by ‘A’ or ‘T’ mononucleotide repeats, which refects a biased base composition with an overall A-T richness in the chloroplast genomes [34–36]. The SSR distribution in different gene regions and genomic quadripartite structures are uneven, and majority of SSRs located on LSC and intergenic spacer, which might provide more information for selecting effective molecular markers for the detection of intra- and interspecifc polymorphisms.

Boundary shifts of the IR region in chloroplast genomes are hypothesized to be one of the primary drivers of overall chloroplast genome size variation [40, 41]. In this study, the longest size of the *R. typhina* chloroplast genome (accession No. MN866894) was from the contractions or expansion of the gene *ndhF* or pseudogene *ycf1* in IR region into SSC, which was different from other *Rhus* species. The *ycf1* gene in the SSC region was the second largest gene in the plastid genome and encoded a protein of approximately 1,800 amino acids, which was highly variable, while the pseudogene *ycf1* located in the IRb region was conserved. Our current results are consistent with the reports from Asaf et al. [38]. Based on a complete chloroplast genome, IR boundary shifts often lead to gene duplication, loss, and large-scale syntenic rearrangement [42]. However, we didn’t detect these events in our samples.

Comparison of the chloroplast genome sequences was considered as an effective strategy to identify the mutation hotspots, which can be used as the specific DNA barcodes [43]. The detection of variability of hotspots as well as universal barcodes (Table 3) showed that the *ycf4*-*cemA* has the second highest average nucleotide diversity (0.03528) after *ccsA*-*ndhD* region (0.03819), next *ndhF*-*rpl32*-*trnL* (0.03399). Yi et al. found that the *trnC*-*trnD* region provided more parsimony-informative characters than the *trnL*-*trnF* region and *ndhF* gene [18], and Pang et al. treated the region *trnH*-*psbA* as a universal barcode [44]. However, basing on the whole chloroplast genome, we detected fewer parsimony information sites and lower nucleotide diversity in *trnC*-*trnD* and *trnH*-*psbA*, whereas found some other more suitable potential molecular markers (*ycf4*-*cemA*, *ndhF*-*rpl32*-*trnL*, *ccsA*-*ndhD*) than universal barcodes [45]. These potential highly variable chloroplast barcodes will increase, rich and update marker resources, especially for traditional Chinese medicine [46]. Moreover, the divergence of *ycf4*-*cemA*, *ndhF*-*rpl32*-*trnL* and *ccsA*-*ndhD* gene was high and majority happened in introns and intergentic spacer, which seemly associated with the results that microsatellites major concentrated intergenic spacer (Figs. 4 and 5). All in all, they both reflected that the interspecific variation were greater than the intraspecific variation, and molecular marker could be helpful for species identification.

The non-synonymous (Ka) and synonymous (Ks) nucleotide substitution patterns of gene are important indicators of gene evolution [47]. The Ka/Ks ratio is usually used to assess whether there are selective pressures on protein-coding gene or to evaluate the rate of gene divergence. Ka/Ks ratios indicate that the gene has undergone positive selection (> 1), neutral selection (close to 1), or purifying seletion (< 1). In our study, the Ka/Ks ratios of *ycf2* vary dramatically in species, i.e., the *ycf2* gene of American individuals (accession Nos. OR800752, OR800753 and OR773067) is undergoing strongly purifying seletion, while for other individuals, the Ka/Ks ratios of *ycf2* gene > 1, undergoing positive selection. We will select a broader group of species to analyze Ks and Ka substitution rate, especially *R. typhina* introduced in China.

Our phylogenetic analyses (Fig. 7) actually supported three major clades, and the broad relationships of Anacardioideae and Spondiadoideae are consistent with the phylogenetic analyses [3, 31, 48]. The genus *Rhus* had a controversial generic limits with *Toxicodendron*, sometimes included as part of *Rhus* [4]. Our results clearly supported that *Toxicodendron* was distinct from *Rhus*, and *Rhus* was more closely related to the *Cotinus*-*Pistacia* clade than to *Toxicodendron*, supporting the narrower generic limit of the genus *Rhus* [17]. In addition, *R. typhina* (OR773067) from US didin’t preferentially cluster with other *R. typhina* individuals from China (MN866894 and MT083895), while grouped with *R. glabra* collected in North American.

Clarifying the phylogenetic positions of *R. glabra* and *R. typhina* are important for understanding the evolution of *Rhus* and the biodiversity assembly in the context of the co-evolution of the *Rhus*-gall aphids and its host plants. Two phylogenetic studies [17, 18] have focused on the genus *Rhus* with a broad sampling, and the results showed incongruence between the nuclear and chloroplast phylogenies, i.e., *R. glabra* appears as sister to *R. typhina* in a clade, sistering to *R. michauxii* according to the nuclear ITS region, whereas *R. glabra* and *R. michauxii* constitute a clade, sistering to *R. typhina* by the chloroplast DNA sequences, and they interpreted such incongruence as evidence for hybridization. One individual of *R. typhina* (OR773067) clustered with *R. glabra* indivuduals might be from the natural hybrids, which has been detected between *R. glabra* and *R. typhina* [49]. Also, Natural hybridization has been reported frequently for *Rhus* species [50]. We will expand the taxon sampling at the genome scale incluing *R*. *michauxii* and further analyze its evolutionary process on the association with the *Rhus*-gall aphid *Melaphis*.

The present results may provide valuable sequence information for molecular phylogenetics and aid in the development of molecular markers for genus *Rhus* and evolutionary analyses of the biological interactions of *Rhus* and the *Rhus*-gall aphids. It will also offer a theoretical basis for resource utilization and conservation of the *Rhus* germplasms.

## Conclusion

In this study, we assemblyed three chloroplast genomes of *Rhus* and identified their gene content and general features, which are both a canonical quadripartite structure. Compared with other *Rhus* species, the interspecific variation were greater than the intraspecific variation, and molecular marker could be helpful for species identification. We detected three sequence variation hotspots (*ycf4*-*cemA*, *ndhF*-*rpl32*-*trnL*, *ccsA*-*ndhD*) which have more parsimony information sites and nucleotide diversity than universal barcodes (*trnC*-*trnD*, *trnL*-*trnF*), which will potentially provide chloroplast markers for further taxonomic, phylogenetic, and population genetic studies in the *Rhus* genus. In *Rhus* genus, the average Ka/Ks ratio was 0.267, which dedicated protein-coding genes of *Rhus* genus was undergoing purifying seletion pressure. And *cemA* and *ycf2* gene are important indicators of gene evolution. The phylogenomic analyses supported *R*. *glabra* was sister to *R*. *typhina*. The present results may provide valuable sequence information for molecular phylogenetics and aid in the development of molecular markers for genus *Rhus* and evolutionary analyses of the biological interactions of *Rhus* and the *Rhus*-gall aphids.

## Methods

### Sample, DNA extraction and sequencing

We collected the fresh leaves of *Rhus glabra* and *R. typhina* in USA in August, 2015. Two *R. glabra* accessions (Voucher nos. Ren_P3002 and Ren_P3051) were from Cincinnati in Ohio and Mansfield in Georgia, respectively, and the *R. typhina* sample (Voucher no. Ren_P3053) from New York. The leaf samples were put in silica gel prior to DNA extraction and the specimen was stored at the Herbarium of School of Life Science, Shanxi University, China. We extracted the total genomic DNA from the leaves by the modified CTAB method [51], and sent the DNAs to the Genomic Sequencing and Analysis Facility (GSAF), (Shanghai, China) for library construction and sequencing on an Illumina HiSeq 4000 platform [52]. The paired-end (PE) reads of 2 × 150 bp (insert size of 400 bp) were generated and the sequencing reads were trimmed for obtained high quality reads [53].

### Chloroplast genome assembly and annotation

After quality trimming, high-quality clean reads were reference-based assembled by the programs GetOrganelle [54] with kmers 21, 45, 65, 85, and 105. The genome annotation was conducted by using Plastid Genome Annotator (PGA) [55], with manual correction using Geneious software (version 11.0.3) [56]. For unannotated or ambiguous region, BLAST in GenBank searched homologous sequences to attempt annotate these regions.

### General characteristics of the chloroplast genome of ***Rhus***

The total chloroplast genome lengths, gene numbers, gene sizes, nucleotide compositions and the lengths of exons and introns were calculate with Geneious (version 11.0.3). The chloroplast physical mappings were drawn using OGDraw (http://ogdraw.mpimp-golm.mpg.de/index.shtml). In MEGA7.0 [57], we calculated the situation frequency of the relative synonymous codon usage by selecting plant plastid. MIcroSAtellite (MISA) (http://pgrc.ipk-gatersleben.de/misa/) software was employed to identify the simple sequence repeats (SSR), and tandem repeats of 1–6 nucleotides was considered as microsatellites [58]. The parameters were set as follows: >10 for mononucleotides, > 6 for dinucleotides, > 3 for trinucleotides, > 3 for tetranucleotides, > 3 for pentanucleotides, and > 3 for hexanucleotides.

### Comparasion of chloroplast genomes in ***Rhus***

24 complete chloroplast genomes representing eight genera of Anacardiaceae from GenBank and our current chloroplast genomes were used to analyze the chloroplast genomic variation of Anacardiaceae species. We re-annotated the chloroplast genomes of *Rhus potaninii* (accession Nos. MN866893 and MN866893), *R. punjabensis* (No. MT230555) and *R. typhina* (Nos. MT083895 and MN866894).

The differences between complete chloroplast genome sequences were analyzed, and chloroplast genome structure of these species compared using mVISTA for further analysis [59]. And LSC/ IRb/ SSC/ IRa junctions can be visualized in IRscope online software (http://irscope.shinapps.io/irapp/).

We used the complete chloroplast genomes of *Rhus* genus to detect the hotspots of species divergence, and analyze the nucleotide diversity (Pi) using the DnaSP v5 software using the sliding window method with a step size of 200 bp and the window length of 800 bp [60] and we considered the outlier values as mutational hotspots.

### Synonymous (Ks) and nonsynonymous (Ka) substitution rate analyses

The DnaSP v5 software [60] was used to estimate substitution rates and Ka/Ks ratios of all 78 protein-coding genes across *Rhus* species (Nos. OP326730, MT230555, MT230556, MN866893, MN866894, MT083895, OR800752, OR800753 and OR7730677). To do this, each protein-coding gene was aligned respectively together with *Pistacia chinensis* (MK738124) as a reference using MAFFT Alignment in Geneious (11.0.3). The parameter settings were as following: Genetic Code: Nuclear Universal. The indication for Ka/Ks “NA” which appears when Ks = 0 (in cases with no substitutions in the alignment, or 100% match) was replaced in all cases with 0.

### Phylogenetic analysis

The phylogenetic position of *R. glabra* and *R. typhina* were assessed using all the protein-coding genes from the complete chloroplast genome sequence of 24 species in the family Anarcadiceae with two species *Boswellia sacra* and *Commiphora gleadensis* from the family Burseraceae as outgroups. Each protein-coding gene was aligned using MAFFT version 7 [61, 62] with Translation Align implemented and then the 78 protein-coding genes formed a dataset by Concatenate Sequence or Alignments in Geneious 10.2.4 with default settings. Maximum-likelihood (ML) analysis was run by RAxML program under the GTRGAMMA model with 1000 bootstrap replicates [63], and the best-fit model for each partition was GTR + G. The phylogenetic tree was visualized by Figtree v1.4.2 (http://tree.bio.ed.ac.uk/software/figtree/).

### Electronic supplementary material

Below is the link to the electronic supplementary material.

**Supplementary Material 1: Fig. S1** Chloroplast genome map of *Rhus glabra* (Accession No. OR800753)

**Supplementary Material 2: Fig. S2** Chloroplast genome map of *Rhus typhina* (Accession No. OR773067)

**Supplementary Material 3: Fig. S3** Sliding-window analysis on the three chloroplast genomes of *Rhus typhina*. Window length: 800 bp; step size: 200 bp; X-axis: position of the midpoint of a window; Y-axis: nucleotide diversity of each window

**Supplementary Material 4: Table S1** Introns and exons of protein coding genes of three *Rhus* chloroplast genomes

**Supplementary Material 5: Table S2** Relative synonymous codon usage (RSCU) of chloroplast genomes of three *Rhus* individuals

## Data Availability

The identified *Rhus glabra* (Voucher nos. Ren_P3002 and Ren_P3051) and *R. typhina* (Voucher no. Ren_P3053) sequences in this study have been submitted to NCBI, and the accession numbers are OR800752, OR800753 and OR773067, respectively. The SRA numbers are SRR26711334, SRR26712902 and SRR26713523, respectively.
