# Supplementary figures and images for: Three complete chloroplast genomes from two north American Rhus species and phylogenomics of Anacardiaceae
Source: BMC Genom Data. 2024 Mar 15;25:30. doi: 10.1186/s12863-024-01200-6 (PMC10943888; doi:10.1186/s12863-024-01200-6)

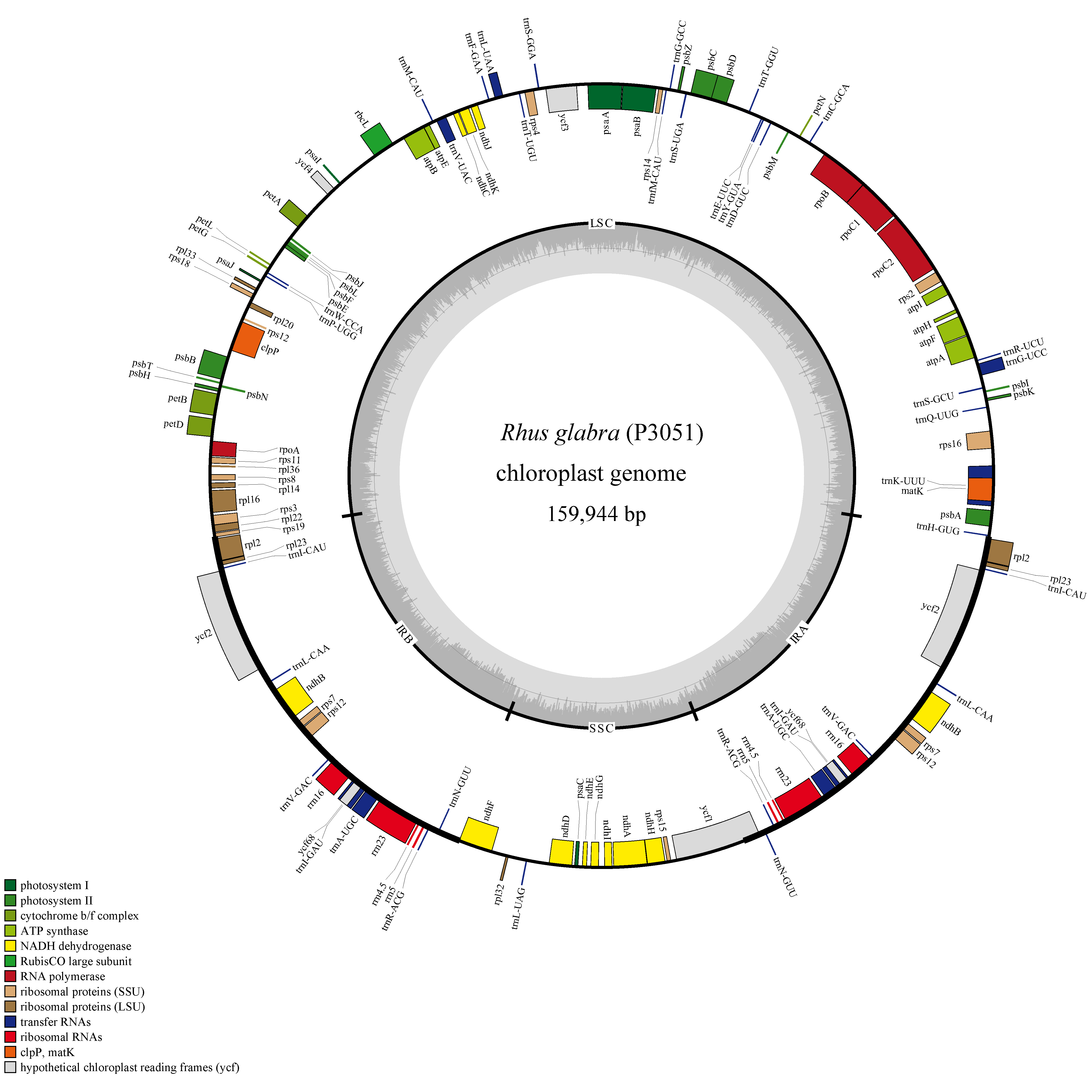

Supplement: Supplementary file 1 — Supplementary Material 1: Fig. S1 Chloroplast genome map of Rhus glabra (Accession No. OR800753) [file 12863_2024_1200_MOESM1_ESM.tif]

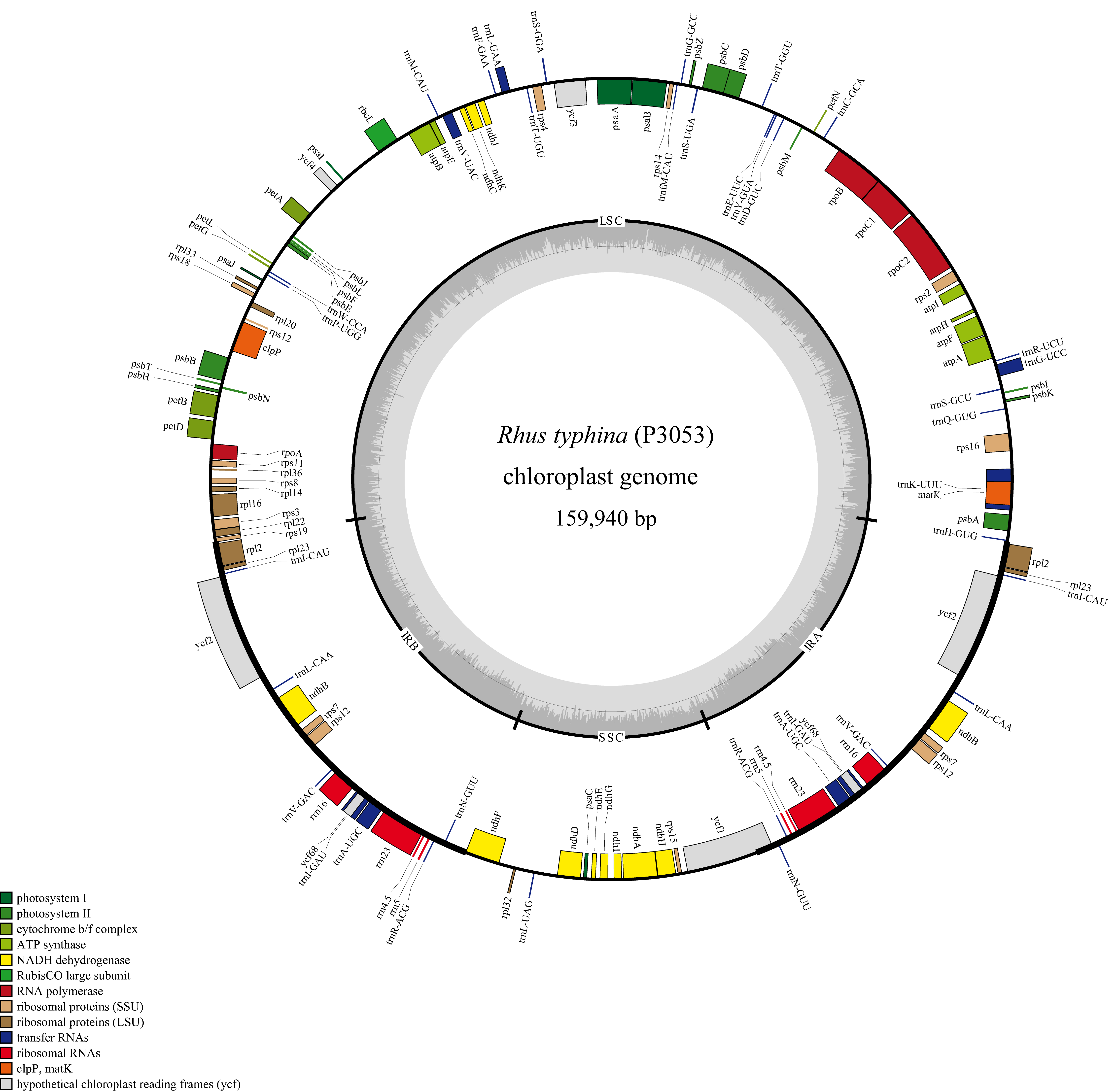

Supplement: Supplementary file 2 — Supplementary Material 2: Fig. S2 Chloroplast genome map of Rhus typhina (Accession No. OR773067) [file 12863_2024_1200_MOESM2_ESM.tif]

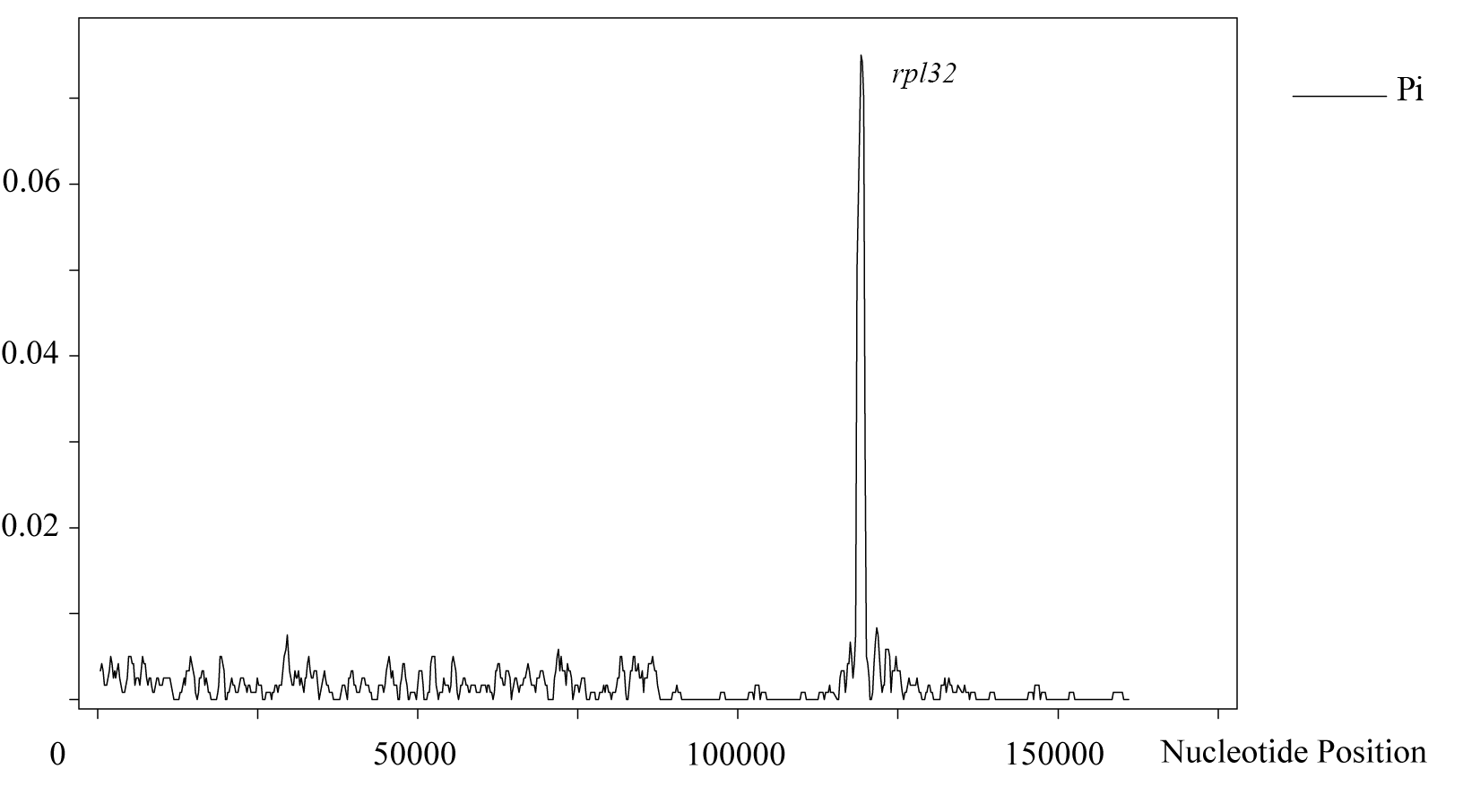

Supplement: Supplementary file 3 — Supplementary Material 3: Fig. S3 Sliding-window analysis on the three chloroplast genomes of Rhus typhina. Window length: 800 bp; step size: 200 bp; X-axis: position of the midpoint of a window; Y-axis: nucleotide diversity of each window [file 12863_2024_1200_MOESM3_ESM.png]
