# Supplementary material for: Three complete chloroplast genomes from two north American Rhus species and phylogenomics of Anacardiaceae
Source: BMC Genom Data. 2024 Mar 15;25:30. doi: 10.1186/s12863-024-01200-6 (PMC10943888; doi:10.1186/s12863-024-01200-6)
Supplement: Supplementary file 4 — Supplementary Material 4: Table S1 Introns and exons of protein coding genes of three Rhus chloroplast genomes [file 12863_2024_1200_MOESM4_ESM.docx]

Table S1 Introns and exons of protein coding genes of three *Rhus* chloroplast genomes

| Gene | Exon I (bp) | | | Intron I(bp) | | | Exon II (bp) | | | Intron II (bp) | | | Exon III (bp) | | |
| --- | --- | --- | --- | --- | --- | --- | --- | --- | --- | --- | --- | --- | --- | --- | --- |
|  | OR800752 | OR800753 | OR773067 | OR800752 | OR800753 | OR773067 | OR800752 | OR800753 | OR773067 | OR800752 | OR800753 | OR773067 | OR800752 | OR800753 | OR773067 |
| *rps16* | 40 | 40 | 40 | 898 | 895 | 895 | 227 | 227 | 227 |  |  |  |  |  |  |
| *atpF* | 145 | 145 | 145 | 757 | 757 | 757 | 410 | 410 | 410 |  |  |  |  |  |  |
| *rpoC1* | 453 | 453 | 453 | 772 | 772 | 772 | 1617 | 1617 | 1617 |  |  |  |  |  |  |
| *ycf3* | 126 | 126 | 126 | 733 | 733 | 733 | 228 | 228 | 228 | 799 | 800 | 799 | 153 | 153 | 153 |
| *clpP* | 71 | 71 | 71 | 784 | 783 | 784 | 292 | 292 | 292 | 641 | 641 | 641 | 228 | 228 | 228 |
| *petB* | 6 | 6 | 6 | 786 | 786 | 786 | 642 | 642 | 642 |  |  |  |  |  |  |
| *petD* | 8 | 8 | 8 | 695 | 695 | 695 | 475 | 475 | 475 |  |  |  |  |  |  |
| *rpl16* | 9 | 9 | 9 | 1059 | 1059 | 1059 | 399 | 399 | 399 |  |  |  |  |  |  |
| *rpl2* | 391 | 391 | 391 | 665 | 665 | 665 | 434 | 434 | 434 |  |  |  |  |  |  |
| *ndhA* | 552 | 552 | 552 | 1118 | 1117 | 1117 | 540 | 540 | 540 |  |  |  |  |  |  |
| *ndhB* | 723 | 723 | 723 | 681 | 681 | 681 | 756 | 756 | 756 |  |  |  |  |  |  |
| *trnV-UAC* | 39 | 39 | 39 | 593 | 539 | 593 | 35 | 35 | 35 |  |  |  |  |  |  |
| *trnL-UAA* | 35 | 35 | 35 | 469 | 469 | 469 | 85 | 50 | 50 |  |  |  |  |  |  |
| *trnK-UUU* | 37 | 37 | 37 | 2598 | 2598 | 2598 | 35 | 35 | 35 |  |  |  |  |  |  |
| *trnG-UCC* | 23 | 23 | 23 | 713 | 713 | 713 | 48 | 48 | 48 |  |  |  |  |  |  |
| *trnI-GAU* | 37 | 37 | 37 | 952 | 952 | 952 | 35 | 35 | 35 |  |  |  |  |  |  |
| *trnA-UGC* | 38 | 38 | 38 | 841 | 841 | 841 | 35 | 35 | 35 |  |  |  |  |  |  |
