# Supplementary material for: Three complete chloroplast genomes from two north American Rhus species and phylogenomics of Anacardiaceae
Source: BMC Genom Data. 2024 Mar 15;25:30. doi: 10.1186/s12863-024-01200-6 (PMC10943888; doi:10.1186/s12863-024-01200-6)
Supplement: Supplementary file 5 — Supplementary Material 5: Table S2 Relative synonymous codon usage (RSCU) of chloroplast genomes of three Rhus individuals [file 12863_2024_1200_MOESM5_ESM.docx]

Table S2 Relative synonymous codon usage (RSCU) of chloroplast genomes of three *Rhus* individuals

| AA | Codon | OR800752 | | OR800753 | | OR773067 | | MN866894 | | MT083895 | |
| --- | --- | --- | --- | --- | --- | --- | --- | --- | --- | --- | --- |
|  |  | NO. | RSCU | NO. | RSCU | NO. | RSCU | NO. | RSCU | NO. | RSCU |
| Phe | UUU(F) | 981 | **1.27** | 982 | **1.28** | 982 | **1.28** | 970 | **1.27** | 983 | **1.27** |
|  | UUC(F) | 558 | 0.73 | 557 | 0.72 | 557 | 0.72 | 556 | 0.73 | 559 | 0.73 |
| Leu | UUA(L) | 823 | **1.75** | 824 | **1.75** | 824 | **1.75** | 825 | **1.76** | 829 | **1.76** |
|  | UUG(L) | 568 | **1.21** | 567 | **1.21** | 567 | **1.21** | 566 | 1.21 | 564 | 1.2 |
|  | CUU(L) | 589 | **1.25** | 591 | **1.26** | 591 | **1.26** | 585 | **1.25** | 589 | **1.25** |
|  | CUC(L) | 214 | 0.46 | 214 | 0.46 | 214 | 0.46 | 219 | 0.47 | 220 | 0.47 |
|  | CUA(L) | 409 | 0.87 | 409 | 0.87 | 409 | 0.87 | 407 | 0.87 | 409 | 0.87 |
|  | CUG(L) | 213 | 0.45 | 213 | 0.45 | 213 | 0.45 | 214 | 0.46 | 213 | 0.45 |
| Ile | AUU(I) | 1098 | **1.47** | 1098 | **1.47** | 1098 | **1.47** | 1103 | **1.47** | 1100 | **1.47** |
|  | AUC(I) | 474 | 0.63 | 474 | 0.63 | 474 | 0.63 | 474 | 0.63 | 477 | 0.64 |
|  | AUA(I) | 676 | 0.9 | 676 | 0.9 | 676 | 0.9 | 671 | 0.9 | 671 | 0.9 |
| Met | AUG(M) | 618 | 1 | 617 | 1 | 617 | 1 | 619 | 1 | 621 | 1 |
| Val | GUU(V) | 524 | **1.45** | 524 | **1.45** | 523 | **1.45** | 524 | **1.45** | 522 | **1.45** |
|  | GUC(V) | 180 | 0.5 | 180 | 0.5 | 181 | 0.5 | 180 | 0.5 | 180 | 0.5 |
|  | GUA(V) | 541 | **1.5** | 540 | **1.5** | 540 | **1.5** | 543 | **1.5** | 545 | **1.51** |
|  | GUG(V) | 196 | 0.54 | 197 | 0.55 | 197 | 0.55 | 197 | 0.55 | 196 | 0.54 |
| Ser | UCU(S) | 570 | **1.63** | 569 | **1.63** | 569 | **1.63** | 568 | **1.62** | 571 | **1.63** |
|  | UCC(S) | 365 | **1.04** | 365 | **1.04** | 365 | **1.04** | 368 | **1.05** | 366 | **1.04** |
|  | UCA(S) | 415 | **1.19** | 416 | **1.19** | 416 | **1.19** | 421 | **1.2** | 415 | **1.18** |
|  | UCG(S) | 203 | 0.58 | 204 | 0.58 | 204 | 0.58 | 205 | 0.58 | 204 | 0.58 |
|  | AGU(S) | 415 | **1.19** | 415 | **1.19** | 415 | **1.19** | 412 | **1.17** | 414 | **1.18** |
|  | AGC(S) | 130 | 0.37 | 130 | 0.37 | 130 | 0.37 | 133 | 0.38 | 132 | 0.38 |
| Pro | CCU(P) | 420 | **1.5** | 420 | **1.5** | 421 | **1.5** | 416 | **1.49** | 419 | **1.5** |
|  | CCC(P) | 220 | 0.79 | 220 | 0.79 | 220 | 0.79 | 221 | 0.79 | 220 | 0.79 |
|  | CCA(P) | 320 | **1.14** | 320 | **1.14** | 320 | **1.14** | 316 | **1.13** | 317 | **1.14** |
|  | CCG(P) | 160 | 0.57 | 160 | 0.57 | 160 | 0.57 | 165 | 0.59 | 161 | 0.58 |
| Thr | ACU(T) | 534 | **1.59** | 534 | **1.58** | 534 | **1.58** | 532 | **1.58** | 536 | **1.59** |
|  | ACC(T) | 256 | 0.76 | 257 | 0.76 | 257 | 0.76 | 257 | 0.77 | 258 | 0.76 |
|  | ACA(T) | 397 | **1.18** | 397 | **1.18** | 397 | **1.18** | 397 | **1.18** | 396 | **1.17** |
|  | ACG(T) | 160 | 0.48 | 161 | 0.48 | 161 | 0.48 | 157 | 0.47 | 161 | 0.48 |
| Ala | GCU(A) | 641 | **1.78** | 642 | **1.78** | 641 | **1.78** | 638 | **1.78** | 637 | **1.77** |
|  | GCC(A) | 229 | 0.64 | 229 | 0.64 | 229 | 0.64 | 224 | 0.63 | 227 | 0.63 |
|  | GCA(A) | 386 | **1.07** | 386 | **1.07** | 386 | **1.07** | 386 | **1.08** | 388 | **1.08** |
|  | GCG(A) | 184 | 0.51 | 184 | 0.51 | 184 | 0.51 | 183 | 0.51 | 185 | 0.51 |
| Tyr | UAU(Y) | 772 | **1.6** | 773 | **1.61** | 773 | **1.61** | 768 | **1.61** | 775 | **1.61** |
|  | UAC(Y) | 191 | 0.4 | 190 | 0.39 | 190 | 0.39 | 187 | 0.39 | 190 | 0.39 |
| His | CAU(H) | 487 | **1.46** | 487 | **1.46** | 487 | **1.46** | 484 | **1.47** | 486 | **1.46** |
|  | CAC(H) | 178 | 0.54 | 178 | 0.54 | 178 | 0.54 | 176 | 0.53 | 179 | 0.54 |
| Gln | CAA(Q) | 714 | **1.53** | 714 | **1.53** | 714 | **1.53** | 708 | **1.53** | 713 | **1.52** |
|  | CAG(Q) | 219 | 0.47 | 219 | 0.47 | 219 | 0.47 | 220 | 0.47 | 223 | 0.48 |
| Asn | AAU(N) | 974 | **1.53** | 973 | **1.53** | 973 | **1.53** | 963 | **1.52** | 973 | **1.52** |
|  | AAC(N) | 303 | 0.47 | 303 | 0.47 | 303 | 0.47 | 305 | 0.48 | 307 | 0.48 |
| Lys | AAA(K) | 1045 | **1.47** | 1047 | **1.47** | 1047 | **1.47** | 1033 | **1.47** | 1050 | **1.47** |
|  | AAG(K) | 375 | 0.53 | 374 | 0.53 | 374 | 0.53 | 374 | 0.53 | 374 | 0.53 |
| Asp | GAU(D) | 866 | **1.57** | 867 | **1.57** | 867 | **1.57** | 861 | **1.57** | 866 | **1.57** |
|  | GAC(D) | 236 | 0.43 | 236 | 0.43 | 236 | 0.43 | 237 | 0.43 | 235 | 0.43 |
| Glu | GAA(E) | 1036 | **1.49** | 1036 | **1.49** | 1036 | **1.49** | 1025 | **1.49** | 1035 | **1.49** |
|  | GAG(E) | 351 | 0.51 | 351 | 0.51 | 351 | 0.51 | 350 | 0.51 | 351 | 0.51 |
| Cys | UGU(C) | 233 | **1.47** | 233 | **1.47** | 233 | **1.47** | 231 | **1.47** | 230 | **1.47** |
|  | UGC(C) | 83 | 0.53 | 83 | 0.53 | 83 | 0.53 | 83 | 0.53 | 83 | 0.53 |
| Trp | UGG(W) | 460 | 1 | 460 | 1 | 460 | 1 | 457 | 1 | 461 | 1 |
| Arg | CGU(R) | 336 | **1.21** | 336 | **1.21** | 336 | **1.21** | 334 | **1.21** | 336 | **1.21** |
|  | CGC(R) | 125 | 0.45 | 125 | 0.45 | 125 | 0.45 | 127 | 0.46 | 125 | 0.45 |
|  | CGA(R) | 377 | **1.36** | 377 | **1.36** | 377 | **1.36** | 373 | **1.35** | 378 | **1.36** |
|  | CGG(R) | 135 | 0.49 | 135 | 0.49 | 135 | 0.49 | 138 | 0.5 | 138 | 0.5 |
|  | AGA(R) | 510 | **1.84** | 510 | **1.84** | 510 | **1.84** | 508 | **1.84** | 507 | **1.83** |
|  | AGG(R) | 180 | 0.65 | 181 | 0.65 | 181 | 0.65 | 179 | 0.65 | 179 | 0.65 |
| Gly | GGU(G) | 603 | **1.31** | 604 | **1.31** | 604 | **1.31** | 601 | **1.31** | 600 | **1.3** |
|  | GGC(G) | 171 | 0.37 | 170 | 0.37 | 170 | 0.37 | 171 | 0.37 | 171 | 0.37 |
|  | GGA(G) | 733 | **1.59** | 734 | **1.6** | 733 | **1.59** | 734 | **1.6** | 737 | **1.6** |
|  | GGG(G) | 333 | 0.72 | 332 | 0.72 | 333 | 0.72 | 333 | 0.72 | 332 | 0.72 |
| Stop | UAA (*) | 50 | **1.7** | 51 | **1.74** | 51 | **1.74** | 49 | **1.67** | 50 | **1.7** |
|  | UGA (*) | 21 | 0.72 | 20 | 0.68 | 20 | 0.68 | 20 | 0.68 | 20 | 0.68 |
|  | UAG (*) | 17 | 0.58 | 17 | 0.58 | 17 | 0.58 | 19 | 0.65 | 18 | 0.61 |
| Total | - | 26781 | - | 26788 | - | 26788 | - | 26700 | - | 26807 | - |

Notes: AA represents amino acids; the species of accessions numbers OR800752 and OR800753 is *R. glabra*; the species of OR773067, MN866894 and MT083895 is *R. typhina*; RSCU values>1 was shown in bold and the codon with* is termination codon.
